# Generation and validation of pX-UASTattB for dose-dependent misexpression studies in *Drosophila*

**DOI:** 10.17912/micropub.biology.000626

**Published:** 2022-08-12

**Authors:** Monika Singh, Jung Hwan Kim

**Affiliations:** 1 Department of Biology, University of Nevada, Reno, NV 89557, USA

## Abstract

The GAL4-UAS binary gene expression system has benefited genetic studies tremendously. However, tools for effective control over the expression levels of transgenes are largely limited. We report a new series pUASTattB-based plasmids. These plasmids preserve the features of pUASTattB but contain a varying number of UAS sites. The expression levels and biological outcomes of a transgene showed a dosage-dependency with the number of UAS sites when using *Dscam1* as a transgene and axon arborization of *Drosophila* sensory neurons as a biological function. Our new plasmids provide novel and useful tools for *Drosophila* genetic studies.

**Figure 1. Generation and validation of pX-UASTattB:**
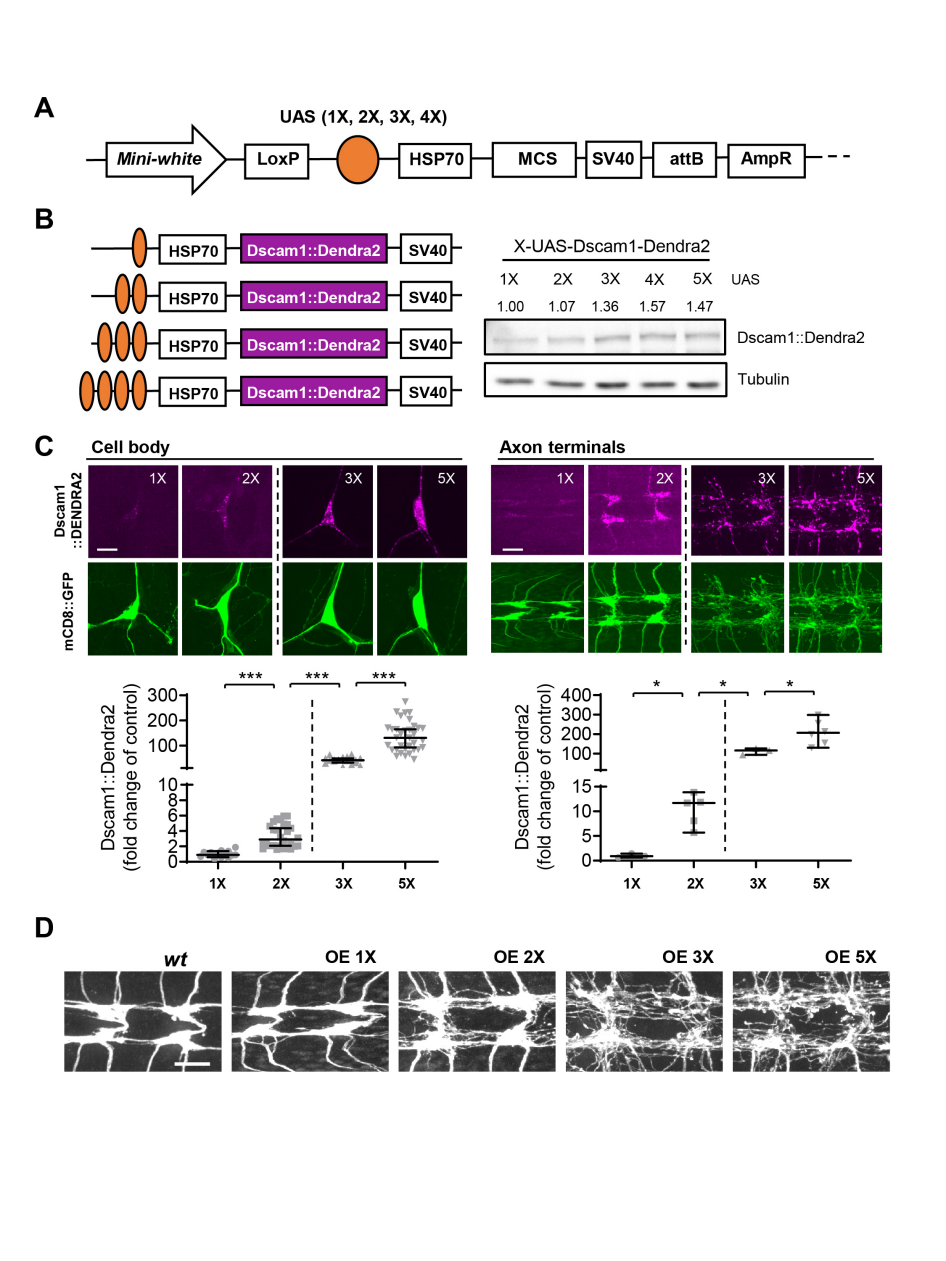
**A.** A schematic of pX-UASTattB. The pX-UASTattB preserves the elements in pUASTattB: HSP70 (hsp70 minimal promoter), MCS (multicloning sites), SV40 (SV40 polyadenylation signal), attB, and AmpR (Ampicillin resistance). **B.** pX-UASTattB constructs driving Dscam1::Dendra2 expression in *Drosophila* S2 cells **.** The coding region of Dscam1::Dendra2 was cloned into p1X-UASTattB, p2X-UASTattB, p3X-UASTattB, p4X-UASTattB, and pUASTattB (5XUAS) respectively (left) and transfected into *Drosophila* S2 cells. Total cell lysates were subjected to SDS-PAGE following Western blot analysis. Tubulin blot was shown as a loading control. The densitometry analysis was performed and shown as a fold change of 1XUAS-Dscam1::Dendra2 above each lane. **C and D.** The transgenic lines of *1X-UAS-Dscam1::Dendra2* , *2X-UAS-Dscam1::Dendra2* , *3X-UAS-Dscam1::Dendra2* , and *5X-UAS-Dscam1::Dendra2* were generated and expressed in *Drosophila* larval class IV dendritic arborization (C4da) neurons using *ppk-GAL4* . The expression levels of Dscam1::Dendra2 (magenta) in the cell body and in the axon terminals were measured and normalized by mCD8::GFP expression (green). Data were expressed as median ± 95% CI (Cell-body: 1X vs 2X, p < 0.0001; 2X vs 3X, p < 0.0001; 3X vs 5X, p < 0.0001 and Axon-terminals: 1X vs 2X p = 0.0357; 2X vs 3X, p = 0.0357; 3X vs 5X, p = 0.0238, two-tailed Mann Whitney test). Note that two different display settings were used for 1X and 2X, 3X and 5X UAS-Dscam1::Dendra2 images and graphs to capture dynamic ranges (C). The axon terminals from C4da neurons that correspond to abdominal segments 4 and 5 were visualized with *mCD8::GFP* using *ppk* -Gal4 to access axon arborization (D). Scale bar = 10 μm.

## Description

The GAL4 (galactose-responsive transcription factor) - UAS (galactose upstream activating sequence) binary gene expression system is originated from fermenting yeast and is composed of a transcription factor GAL4 and a 17-nucleotide DNA sequence of UAS (Duffy, 2002; Southall et al., 2008). The simple and specific interaction between GAL4 and UAS made the system ideal for cell-type and tissue-specific transgene expression. Since the initial adoption by Fisher et al. (Fischer et al., 1988) and with the past and on-going community-wide effort of generating GAL4 lines, the GAL4-UAS binary system revolutionized *Drosophila* genetics (Jenett et al., 2012; Southall et al., 2008).

However, few tools are available for fine-tuning the expression levels of transgenes, often expressing transgenes at an excessive amount. Traditionally, this was circumvented by using transgenic lines from different genomic insertions, hence different expression levels. However, this can potentially complicate experimental setups because a genomic insertion of a transgene can disrupt endogenous gene expression from the inserted locus. Additionally, this precludes a study for a dosage-dependent effect because different transgenic insertions affect endogenous loci differentially. Ideally, a tool should provide differential expression of transgenes without such complications. A previous work by Pfeiffer extensively refined targeted gene expression tools using green fluorescent protein as a reporter and showed a possibility of modulating transgene expression by changing the number of UAS sites (Pfeiffer et al., 2010). However, only a limited number of plasmids was available especially for lower transgene expression and quantitative measurement was not provided.

To overcome these limitations, we have generated a series of new plasmids that is based on the popularly used pUASTattB plasmid (Bischof et al., 2007). We named these pX-UASTattB ( **Figure 1A** ). The pX-UASTattB contains one to four UAS sequences instead of five from pUASTattB without changing other features of pUASTattB. The pX-UASTattB provides an attB site for targeted transgene insertion as in pUASTattB. The pX-UASTattB also contains the varying number of UAS sequences followed by hsp70 promoter (HSP70) for GAL4-induced gene expression, a multicloning sites (MCS) for readily available cloning, and SV40 polyadenylation signal (SV40) for transcription termination and polyadenylation as in pUASTattB and pUAST (Brand and Perrimon, 1993). These plasmids also offer an ampicillin-resistant gene (AmpR) for cloning purposes and a mini-white gene for phenotypic screening ( **Figure 1A** ). The pX-UASTattB was designed for transgene expression from an identical genomic landing site but with differential transgene expression levels depending on the number of UAS sites.

To verify that pX-UASTattB can be utilized for dose-dependent expression of a transgene, we built DNA constructs using Down syndrome cell adhesion molecule ( *Dscam1* ) in pX-UASTattB and pUASTattB backbone. These provide 1X, 2X, 3X, 4X, and 5X UAS sequences for transgene expression. Dscam1 is a neuronal cell adhesion molecule. A dosage-dependent role of Dscam1 expression levels was suggested in presynaptic arborization (Kim et al., 2013). Therefore, we choose Dscam1 as a transgene to test. The Dscam1 constructs were tagged with Dendra2 fluorescent protein for visualizing transgene expression levels. We first tested whether these fewer numbers of UAS sequences can drive GAL4-dependent gene expression in vitro. The Dscam1::Dendra2 DNA constructs that contain one to five UAS sequences were transfected into cultured *Drosophila* S2 cells along with tubP-GAL4 (Lee and Luo, 1999). Total cell lysates were resolved by SDS-PAGE and subjected to Western blot analysis using Dendra2 antibody. Tubulin antibody was used for a loading control. We found that all Dscam1::Dendra2 DNA constructs were able to drive the Dscam1::Dendra2 expression ( **Figure 1B** ). Although the differences were not significantly big, it showed an increasing trend of transgene expression as the number of UAS sites increase.

Next, we selected 1X, 2X, 3X, and 5XUAS-Dscam1::Dendra2 and generated *Dscam1::Dendra2* transgenic fly lines using an identical attP landing site (Bischof et al., 2007). Transgenes were expressed in *Drosophila* larval class IV dendritic arborization (C4da) neurons along with a membrane marker, mCD8::GFP using *ppk-GAL4* (Grueber et al., 2007). After immunostaining, the expression levels of Dscam1::Dendra2 were measured from C4da cell bodies and from C4da axon terminals ( **Figure 1C** ). The expression levels of mCD8::GFP were used as a normalization control. The result showed dramatically dynamic transgene expression levels that were ranging two-orders of magnitude. When normalized by the expression levels of *1XUAS-Dscam1::Dendra2* , a 3-fold increase by *2X* , a 41-fold by *3X* , and a 136-fold by *5XUAS-Dscam1::Dendra2* . We observed the dynamic ranges with a similar trend but with a difference in the axon terminals — a 10-fold by *2X* , a 112-fold by *3X* , a 219-fold by *5X* as compared to *1XUAS-Dscam1::Dendra2* . Dscam1 expression levels correlates with the sizes of presynaptic arbors (Kim et al., 2013). We examined axon arborization of the C4da neurons that express Dscam1::Dendra2 from different numbers of UAS ( **Figure 1D** ). The connecting fibers between each C4da neuropil in the ventral nerve cord provide a simple way of evaluating axon arborization (Sterne et al., 2015; Wang et al., 2013). It normally does not exceed 2-3 fibers as shown in a wild-type control ( **Figure 1D** ). We observed no difference in C4da neurons expressing 1XUAS-Dscam1::Dendra2. However, an increasing number of connecting fibers were observed as the number of UAS sites increase — as Dscam1::Dendra2 expression levels increase. Taken together, these clearly show that pX-UASTattB provides a dynamic range of transgene expression levels in vivo and provides a useful tool to study dosage-dependent gene functions in vivo.

## Methods

***Drosophila* *melanogaster* strains**

*Drosophila* strains were kept under standard condition at 25 ⁰C in a humidified chamber. The following strains were used in this study: *w ^1118^* (Bloomington Drosophila Stock center, Bloomington, IN, stock number: 3605), *ppk-GAL4* (Grueber et al., 2007).

**DNA constructs and *Drosophila* transgenic flies**

The double-strand DNA sequences for UAS sequences and the Hsp70 minimal promoter were chemically synthesized and cloned into pBluescript II SK(+) using HindIII and EcoRI by GenScript (Piscataway, NJ). The UAS sequences and *hsp70* minimal promoter were excised and ligated into pUASTattB plasmid using HindIII and EcoRI. The resulting pX-UASTattB plasmids were verified by Sanger sequencing. The coding sequence of *Dscam1* with an ectodomain isoform (4.3-6.36-9.25-17.2) (Kim et al., 2013) was used to generate the *X-UAS-Dscam1-Dendra2* constructs.

Transgenic flies were generated by PhiC31-mediated germline transformation (Bischof et al., 2007) using attP40 as a landing site.

**S2 cell culture, transfection, and Western blot analysis**

S2 cells were maintained in *Drosophila* Schneider’s medium (ThermoFisher, Waltham, MA) supplemented with 10% fetal bovine serum (Sigma, St. Louis, MO) at 27ºC in a humidified incubator. Cells were transfected with indicated DNA constructs along with tubulinP-Gal4 (Lee and Luo, 2001) using Polyethylenimine method (Longo et al., 2013). For Western blot analysis, S2 cells were washed two times with PBS. The total lysates were resolved on 8% SDS-PAGE gels and subjected to Western blot analysis as previously described (Kim et al., 2013). Primary antibodies used were rabbit polyclonal anti-Dendra2 (Antibodies-online, Limerick, PA) and mouse monoclonal anti-tubulin (DM1A) (Sigma, St. Louis, MO).

**Immunostaining**

*Drosophila* third-instar larvae were prepared as previously described (Kim et al., 2013). Primary antibodies used were chicken polyclonal anti-GFP (Aves Labs, Tigard, OR), rabbit polyclonal anti-Dendra2 (Antibodies-online, Limerick, PA), The secondary antibodies used were Cy2-conjugated goat anti-chicken and Cy5-conjugated goat anti-Rabbit (from the Jackson ImmunoResearch, West Grove, PA). The imaging was done with a custom-built spinning disk confocal microscope equipped with a 63x oil-immersion objective with a 0.3 µm step-sizes. The image acquisition was done by Hamamatsu Flash V3 sCMOS camera with a 16-bit image depth to avoid image saturation. The resulting 3D images were projected into 2D images using a maximum projection method in the imageJ software. A region of interest was drawn in the cell body of C4da neurons and in the neuropil area from the ventral nerve cord, and the mean fluorescence intensity was measured using imageJ software to quantify *UAS-Dscam1::Dendra2* transgene expression levels. Since the activity of GAL4 varies among individual neurons, the mean fluorescence intensity of *UAS-mCD8::GFP* was measured from the same region of interest and used as a normalization control.

**Experimental design and statistical analysis**

Statistical analysis was performed as two-tailed using GraphPad Prism version 7.04 (GraphPad Software). The Mann-Whitney test was used. A p value smaller than 0.05 was considered statistically significant. All p values are indicated as NS; non-significant, *; P < 0.05, **; P < 0.01, ***; P < 0.001.

## Reagents

Rabbit polyclonal Dendra2 antibody (AB_2915898), mouse monoclonal Tubulin antibody (AB_477583), chicken polyclonal GFP antibody (AB_2307313), and *Drosophila* S2R+ cells (CVCL_Z831). pX-UASTattB (p1XUASTattB, p2XUASTattB, p3XUASTattB, and p4XUASTattB) are from this study. p4X-UASTattB (Addgene_189858), p3X-UASTattB (Addgene_189859), p2X-UASTattB (Addgene_189860), and p1X-UASTattB (Addgene_189861).
